# Online Medicine for Pregnant Women

**DOI:** 10.1155/2014/379427

**Published:** 2014-07-13

**Authors:** Sharon Davidesko, David Segal, Roni Peleg

**Affiliations:** ^1^Ben-Gurion University of the Negev, P.O. Box 653, 84105 Beer-Sheva, Israel; ^2^Division of Obstetrics and Gynecology, Soroka Medical Center, P.O. Box 151, 84101 Beer-Sheva, Israel; ^3^Women Health Center, Clalit Health Services, Southern District, Henrietta Szold 1, 89428 Beer-Sheva, Israel; ^4^The Department of Family Medicine and Siaal Research Center for Family Practice and Primary Care, Division of Community Health, Faculty of Health Sciences, Ben-Gurion University of the Negev, P.O. Box 653, 84105 Beer-Sheva, Israel

## Abstract

*Objective.* To assess the use of cell phones and email as means of communication between pregnant women and their gynecologists and family physicians. *Study Design.* A cross-sectional study of pregnant women at routine followup. One hundred and twenty women participated in the study. *Results.* The mean age was 27.4 ± 3.4 years. One hundred nineteen women owned a cell phone and 114 (95%) had an email address. Seventy-two women (60%) had their gynecologist's cell phone number and 50 women (42%) had their family physician's cell phone number. More women contacted their gynecologist via cell phone or email during pregnancy compared to their family physician (*P* = 0.005 and 0.009, resp.). Most preferred to communicate with their physician via cell phone at predetermined times, but by email at any time during the day (*P* < 0.0001). They would use cell phones for emergencies or unusual problems but preferred email for other matters (*P* < 0.0001). *Conclusions.* Pregnant women in the Negev region do not have a preference between the use of cell phones or email for medical consultation with their gynecologist or family physician. The provision of the physician's cell phone numbers or email address together with the provision of guidelines and resources could improve healthcare services.

## 1. Introduction

Israel passed a Health Insurance law in 1995 that mandates healthcare services by healthcare funds (HMOs) for the entire population. There is competition among the funds to improve efficiency and provide optimal care to the satisfaction of their patients, while still meeting budgetary constraints. One of the ways to achieve these goals is the use of advanced means of communication such as provision of physicians' cell phone numbers and email addresses to patients for those cases in which this form of communication can make patient-physician communication more efficient. The use of cell phones and email to reduce the work burden of clinic physicians and to improve patient-physician communication has been shown to be effective [1]. Experience has shown that cell phone consultations are more effective than in-person consultations in the clinic [2, 3] especially for the ongoing treatment of chronic diseases [4, 5]. Educated use of this form of consultation enables patients to get counsel when they require it. Cell phone consultations can save travel time as well as waiting time in the clinic [6].

Patients often contact their physicians by cell phone [7]. One study showed that 83.1% of the patients that contacted their physician by cell phone solved their problem and did not have to come to the clinic. In addition, in 52.8% of the cases it was possible to monitor the patients by phone [8]. In another study most of the family physicians surveyed thought that cell phone consultation was of equal value to a face-to-face appointment [9].

Electronic communication is a revolutionary development in healthcare services [10]. The results of a survey, which assessed communication between patients and physicians, showed that patients were satisfied with the option of electronic communication with their physicians. The investigators found that email was a convenient, useful way for physicians to achieve their objectives without any reported problems [11].

In order to evaluate this development in the field of patient-physician communication one should assess the advantages and disadvantages of its use. Proper use of email can improve communication and serve as a primary instrument for consulting in the healthcare system [12]. A study that investigated the use of email for communication with patients found that the main reasons for choosing this mode of communication, among physicians who were satisfied with its use, was that it saved time (33%) and helped provide better care (20%). Among physicians who were not happy with this mode of communication the major reason for its use was that patients requested it (80%) [13]. In another study of communication with patients by email the physicians reported a high degree of satisfaction with this mode of communication [14]. Physicians should be aware of the advantages and disadvantages of electronic communication with patients so as to make the best possible use of it.

Although provision of cell phone numbers [15] or email addresses [13] to patients is simple and can make patient-physician communication easier, it can also increase the physician's work load and have a negative effect on the physician's work environment and even on their free time [16].

Pregnant women comprise a unique population that needs monitoring over the course of pregnancy. Pregnancy entails potential condition-related complications on the one hand while necessitating increased monitoring of chronic diseases that are unrelated to gender or pregnancy on the other. This unique situation requires the professional skills of the gynecologist together with the ongoing care of the family physician. The latter knows the patients and their medical and biopsychosocial circumstances and information that is very important for the decision-making process. The mode of communication with the gynecologist and the family physician is important as well as its availability at times of need under these unique medical circumstances. Over the course of pregnancy women often feel a need to contact their physician about their pregnancy, per se, as well as any causes of concern that may arise or new and troublesome symptoms. To our knowledge no paper has been published to date on patient-physician communication among pregnant women.

### 1.1. Setting

In Israel the treatment and followup of pregnant women are carried out by family physicians as well as obstetricians and gynecologists. Deliveries are performed by obstetricians and midwifes, but not by family physicians. In the current study family physicians serve as a reference group for comparison with obstetricians. In Israel there is a combined residency program for obstetrics and gynecology, so for convenience we use the term gynecologist when referring to either gynecologists or obstetricians.

## 2. Materials and Methods

The primary aim of the study was to evaluate the use of cell phones or email by pregnant women to consult with their gynecologist or family physician and their use of the Internet to search for information on their pregnancy.

The secondary aims of the study were as follows:

- to assess whether pregnant patients have the cell phone number or the email address of their gynecologist or family physician,
- to compare how pregnant women consult with their gynecologist and family physician,
- to evaluate the advantages and disadvantages of these modes of communication,
- to assess the effect of patient age, educational level, and other sociodemographic variables on the preferred mode of communication,
- to assess use of Internet searches to obtain information on pregnancy-related issues,
- to improve our understanding of this new mode of healthcare service.

This was a cross-sectional study. Personal interviews were conducted with Hebrew-speaking pregnant women of 18 years of age or older who came to the Women's Health Center of the Clalit Healthcare Services in Beer-Sheva for a routine pregnancy checkup and agreed to participate in the study. Women with cognitive problems and those who were unable to answer the questionnaire items were not included in the study.

The study instrument was a questionnaire completed by personal interview. The first part covered patient attitudes towards getting their physicians' cell phone number and email address for medical consultations during pregnancy and use of the Internet to obtain medical information. The second part included patient sociodemographic data. The questionnaire was tested in a pilot study with 10 participants and was revised in light of their comments.

Statistical analyses were conducted with the SPSS software package; version 19.0. Statistical tests were used for differences between the two primary study groups. In univariate analyses the Chi-square test was used for categorical variables and *t*-tests for continuous variables. Statistical significance was set at *P* < 0.05.

The Helsinki Committee of the Meir Medical Center approved the study (number 140/2012).

## 3. Results

One hundred and twenty women participated in the study. Their mean age was 27.4 ± 3.4. One hundred and five women (96%) were married and most lived in Beer-Sheva (59%). Ninety-eight women (82%) were born in Israel. The sociodemographic characteristics of the study population are shown in Table 1.

### 3.1. Attitudes towards the Use of Cell Phones for Consultations with Gynecologists and Family Physicians (Table 2)

One hundred and nineteen of the 120 participants have cell phones. Most of the participants were very interested in receiving the cell phone number of their gynecologist and family physician (92.5% and 95%, resp.). Most of them felt that having their physician's cell phone number could improve the quality of their communication (4.53 for their gynecologist and 4.56 for their family physician on a scale from 1 to 5, with 5 representing strong agreement). The women also agreed that having their physician's cell phone number would increase their personal sense of security even if they did not actually contact the physician. The women agreed that calling the physician during work hours could impair the physician's work (4.0 for the gynecologist and 4.05 for the family physician).

In the majority of issues surveyed there were no statistically significant differences in the participants' responses between gynecologists and family physicians. The exceptions were that more women had their gynecologist's number than their family physician's number (*P* = 0.004) and more women actually contacted their gynecologist than their family physician by cell phone during pregnancy (*P* = 0.005).

### 3.2. Attitudes towards the Use of Email for Consultations with Gynecologists and Family Physicians (Table 3)

One hundred and fourteen participants (95%) have email addresses. Most of the women were very interested in getting email addresses from their gynecologist (89.2%) and their family physician (88.3%). Most of them felt that having their physician's cell phone number could improve the quality of their communication (4.6 for their gynecologist and 4.58 for their family physician). Similarly, the women agreed that having their physician's cell phone number would increase their personal sense of security even if they did not actually contact the physician. They thought that having the cell phone number could help solve medical problems and reduce the number of visits to the clinic and emergency room. The women responded that calling the physician during work hours could impair the physician's work to a moderate degree (3.2 for both the gynecologist and the family physician).

The participants thought that there is greater risk of impaired communication through email with their family physician (4.03) than with their gynecologist (3.08) (*P* < 0.0001). More women had their gynecologist's email address (61%) than their family physician's email address (38%) (*P* = 0.005). More women contacted their gynecologist by means of email during pregnancy (34%) than their family physician (9%) (*P* = 0.009).

### 3.3. A Comparison of Attitudes towards Getting the Family Physician's Cell Phone Number or Email Address (Table 4)

There was no statistically significant preference for getting a cell phone number or email address or as to which would be more likely to improve communication with the family physician, provide a greater sense of personal security, or reduce the number of clinic or emergency room visits. More women said that they would prefer to contact their family physician by cell phone at predetermined days or hours compared with any hour of the day by email (*P* < 0.0001). Similarly, women prefer the cell phone to email in unusual circumstances (*P* < 0.001).

### 3.4. A Comparison of Attitudes towards Getting the Gynecologist's Cell Phone Number or Email Address (Table 5)

There was no statistically significant preference for getting a cell phone number or email address or as to which would be more likely to improve communication with the gynecologist, provide a greater sense of personal security, or reduce the number of clinic or emergency room visits. More women said that they would prefer to contact their gynecologist by cell phone at predetermined days or hours compared with any hour of the day by email (*P* < 0.0001). Similarly, women prefer the cell phone to email in unusual circumstances (*P* < 0.0001).

### 3.5. Internet Searches for Information Related to Pregnancy (Table 6)

Most of the women (60%) reported that they conduct Internet searches on pregnancy often but 76% never discussed the information obtained with their gynecologist and 81% never did so with their family physician. The mean age of women who conducted Internet searches on pregnancy was higher than those who did not (*P* < 0.001). Most of the women who live in Beer-Sheva (*N* = 71) conducted Internet searches, while most of those who did not conduct Internet searches live in Bedouin regions (*P* < 0.001) and define themselves as religious (*P* < 0.001). Women who conducted fewer Internet searches or did not conduct them at all have fewer years of education (*P* < 0.001) and are more likely to be unemployed (*P* < 0.001) and have lower mean incomes (*P* = 0.002).

## 4. Discussion

The results of the study show that the participants were interested in receiving the cell phone number or email address of both of their treating physicians without any preference for either modality.

We found that most of the women were very interested in getting the cell phone number and email address of their gynecologist (92.5% and 89.2%, resp.) or family physician (95% and 88.3%, resp.). These findings are in contradiction to the results of other studies conducted in the same geographic region among Jews [17] and Bedouins (personal communication), which found a clear preference for cell phone number over email address. The difference in results may stem from differences in the study populations. Pregnant women are younger on average than the general population so that they may have more experience, knowledge, and access to electronic means of communication. In addition, pregnant women comprise a unique population that requires specific monitoring to prevent complications and to control chronic diseases that are not related to the pregnancy and because the group is composed solely of women. These circumstances necessitate the specific professional skills of gynecologists and the continuity of care and familiarity with patients over time in biopsychosocial terms that the family physician can provide. The mode of communication with patients practiced by gynecologists and family physicians as well as its availability and accessibility are of great importance.

More pregnant women had their gynecologists' cell phone number and/or email address and consulted with their gynecologist by electronic modes than with their family physician over the course of their pregnancy. A possible explanation for this finding is that pregnant women are usually healthy and have little need in general for contact with healthcare services or family physicians, so they turn to gynecologists during pregnancy because they perceive them as the natural address for pregnancy-related issues.

Telephone calls interrupt the routine work of the physician in the clinic, while communication through email does not, because the physician can relate to the patient who sent an email message when they are not with another patient [17]. This is consistent with the findings of our study. Phone calls enable consultations in real time compared with email communication, which does not always elicit an immediate response. In the present study the women were asked about the circumstances under which they would consult with their physician by cell phone or email. The results show that women communicate by cell phone if the circumstances are urgent or unusual. On the other hand they use email for any question that arises. This finding highlights the possibility that patients are able to make reasonable use of varying communication modes for different healthcare needs. The women declared that they make contact with their gynecologist or family physician during all hours of the day by email, but only at predetermined times by cell phone. In another study conducted in a surgical medical center provision of cell phone numbers by the treating physician was interpreted as a sign of caring on the physician's part and patients made use of this service in an efficient manner when they needed it [18]. In general, patients are happier when they have the option of communicating with their physician by personal cell phone [9, 18].

Having the physician's cell phone number and/or email address can give patients a sense of personal security even if they do not actually use it. If used, it can lead to a reduction in clinic and emergency room visits and a decrease in the work burden of physicians in clinics and in the hospital. The results of another study showed that use of email for medical consultation led to a significant reduction in emergency room visits [6]. Even though there have been reports of email consultations from as long ago as a decade, the use of email has become popular later than the use of cell phones [19]. Most of the participants in the present study have email addresses (95%) compared to 85.5% and 22% in previous studies conducted in the same geographical region in Jewish [17] and Bedouin populations (personal communication), respectively. One possible explanation for these differences is that pregnant women are usually younger than patients in family medicine practices who made up the study populations in the earlier studies.

In the present study we found that all the women who did not perform pregnancy-related Internet searches were Bedouin women who defined themselves as religious. This indicates that there are significant differences between the Jewish and Bedouin sectors of the population in terms of use of the Internet and email. This may stem from lower availability and accessibility of computers and other means of electronic communication in the Bedouin sector compared to the Jewish sector.

In a previous study on the attitudes of physicians in the Negev to providing their email addresses to their patients, 65% expressed concern that the absence of a physical examination could lead to misdiagnosis and treatment, 58% stated that in the case of emergency they would recommend that their patients visit the emergency room, and 57% believed that communication through email could impair the quality of care and were concerned about medical negligence suits [20].

In a world in which an increasing number of people have access to the Internet and email and use them for many and varied needs, the provision of healthcare services through electronic communication could become, in the future, a central modality of medical consultation. However, there is a glaring lack of controlled studies supporting this means of communication for medical services or providing information on how to integrate these technologies into the daily work routine of the physician [11].

The use of email for patient-physician communication also raises an important ethical issue. Studies conducted in northern Europe found that medical confidentiality could not be guaranteed using these technologies and as a result hospitals developed computerized systems in which patients can contact their physicians in a secure manner [21, 22].

There are no clear regulations or guidelines in Israel regarding the use of cell phones, email, and social networks such as Facebook and Twitter for medical consultation and physicians use them as they see fit.

Although there are clear advantages to the use of cell phones and email for medical consultation, there are also disadvantages including invasion of the physician's free time beyond their defined and compensated work hours, interruption to the provision of medical care for other patients during clinic work hours, and the risk of mistakes in medical decision making [23]. All the participants in the present study agreed that gynecologists and family physicians should be reimbursed for healthcare provided through cell phones and email. The formulation of guidelines for the use of cell phones and email for medical consultations, such as setting aside dedicated time for this type of patient-physician communication, could improve physicians' willingness to use it [24]. Another possible means of improving medical service for pregnant women could be the inclusion of midwives who specialize in pregnancy monitoring in the team that provides consultation through cell phones and email. This could improve the service since it would reduce costs and improve availability.

This study has several limitations, including the relatively small study population of 120 pregnant women, which may have limited statistical power and led to a type II error. Since the vast majority in the study were very interested in getting the cell phone numbers and email addresses of their gynecologists and family physicians it was not possible to conduct a logistic regression analysis to look for characteristics of the participating women that would predict whether they preferred one modality over the other. This study was conducted in a specific geographical region in women's health clinic of the Negev region of Israel. Since there are significant differences relating to health and pregnancy in different classes and cultures, the results of this study cannot be generalized to all the populations of pregnant women in Israel and around the world. It is also possible that women who did not participate in the study, including women who do not come to the clinic for regular monitoring during pregnancy, could have different attitudes towards the study questions, thus leading to a potential bias.

## 5. Conclusions

Pregnant women in the Negev region do not have a preference between the use of cell phones or email for medical consultation with their gynecologist or family physician. The provision of the physician's cell phone numbers or email address, together with provision of guidelines and resources, could improve healthcare services by reducing clinic and emergency room visits, providing a sense of personal security to patients, and improving the quality of the patient-physician relationship. Understanding the unique advantages and disadvantages of these modes of communication could lead to their effective use in different conditions, including treatment for pregnant women. To this end it is recommended to formulate ethical and legal guidelines relating to the use of cell phones and email for healthcare services.

We hope that the findings of this study will help further this understanding of the use of cell phones and email among pregnant women specifically and in healthcare services in general.

## Figures and Tables

**Table 1:** Sociodemographic and health characteristics of the study population (*N* = 120).

Variable	Result
Age in years	
Mean ± SD	27.4 ± 4.3
Range	18–38
Family status [*N* (%)]	
Single	5 (4)
Married	115 (96)
Place of residence [*N* (%)]	
Beer-Sheva	71 (59)
Nearby city	20 (17)
Agricultural settlement	11 (9)
Bedouin sector	18 (15)
Country of birth [*N* (%)]	
Israel	98 (82)
Former USSR	12 (10)
Europe	5 (4)
USA/Canada	3 (3)
Africa/Asia	2 (2)
Years of education	
Mean ± SD	11.8 ± 0.7
Range	9–14
Present work status [*N* (%)]	
Employed	82 (68)
Student	16 (13)
Unemployed	22 (18)
Income	
Low	63 (53)
Average	43 (36)
High	14 (12)
How would you rate your health condition? [*N* (%)]	
Excellent	81 (68)
Very good	25 (21)
Good	4 (3)
Reasonable	8 (7)
Poor	2 (2)
Do you suffer from a chronic disease? [*N* (%)]	
Yes	12 (10)
No	108 (90)
Population sector? [*N* (%)]	
Jewish	93 (78)
Bedouin	27 (23)
Number of children	
Mean ± SD	1.08 ± 1.31
Range	0–7
Week of pregnancy	
Mean ± SD	20.87 ± 8.05
Range	7–37

**Table 2:** Attitudes towards medical consultation through cell phones.

Variable	Gynecologist	Family physician	*P*
How do you feel about getting your physician's cell phone number? [*N* (%)]			
Very interested	111 (92.5)	114 (95.0)	0.747
Would not object	8 (6.7)	5 (4.2)	
Not interested	1 (0.8)	1 (0.8)	
Do you agree with the following statements regarding getting your physician's cell phone number? (scale of 1–5)			
It could improve the relationship between us:			
Mean ± SD	4.53 ± 0.78	4.58 ± 0.71	0.678
Range	2–5	2–5	
It could improve my sense of security even if I do not use it:			
Mean ± SD	4.53 ± 0.78	4.57 ± 0.71	0.604
Range	2–5	2–5	
The cell phone is an effective mode of communication that could solve my problems:			
Mean ± SD	4.28 ± 0.74	4.37 ± 0.71	0.377
Range	2–5	2–5	
The cell phone can cut down on the number of clinic visits:			
Mean ± SD	4.28 ± 0.74	4.33 ± 0.73	0.598
Range	2–5	2–5	
The cell phone can reduce the number of emergency room visits:			
Mean ± SD	4.28 ± 0.74	4.33 ± 0.74	0.602
Range	2–5	2–5	
At what times would you call the physician? [*N* (%)]			
I do not intend to call	2 (2)	2 (2)	0.994
Only at appointed hours	47 (39)	49 (41)	
Only during daytime hours (except Saturdays and holidays)	56 (47)	54 (45)	
At all hours including nights, Saturdays, and holidays	15 (13)	15 (13)	
Under which circumstance would you call your physician? [*N* (%)]			
I do not intend to call	1 (1)	1 (1)	0.945
Only in unusual circumstances	75 (63)	73 (61)	
For any questions that I think I require a medical consultation	44 (37)	46 (38)	
The physician should not be called because it could interfere with their privacy when they are not working (scale of 1–5):			
Mean ± SD	3.94 ± 0.86	4.03 ± 0.98	0.450
Range	2–5	1–5	
The physician should not be called because there are telephone centers that are active after clinic hours (scale of 1–5):			
Mean ± SD	4.08 ± 0.89	4.20 ± 0.83	0.281
Range	2–5	1–5	
The physician should not be called because in emergencies one can call for an ambulance or go to the emergency room (scale of 1–5):			
Mean ± SD	4.31 ± 0.71	4.23 ± 0.83	0.423
Range	2–5	1–5	
The physician should not be called because medical errors can occur if a physical examination is not performed (scale of 1–5):			
Mean ± SD	3.67 ± 0.99	3.73 ± 1.03	0.646
Range	1–5	1–5	
The physician should not be called because there is a risk of miscommunication (scale of 1 to 5):			
Mean ± SD	3.92 ± 0.94	3.98 ± 0.97	0.627
Range	1–5	1–5	
The physician should not be called because it can interfere with their clinic work (scale of 1 to 5):			
Mean ± SD	4.00 ± 0.90	4.05 ± 1.00	0.684
Range	1–5	1–5	
Have you asked for your physician's cell phone number in the past? [*N* (%)]			
Yes	33 (28)	29 (24)	0.555
Do you have your physician's cell phone number? [*N* (%)]			
Yes	72 (60)	50 (42)	0.004
Have you contacted your physician by cell phone since you became pregnant? [*N* (%)]			
Yes	46 (38)	26 (22)	0.005

**Table 3:** Attitudes towards medical consultation through email.

Variable	Gynecologist	Family physician	*P*
How do you feel about getting your physician's email address? [*N* (%)]			
Very interested	107 (89.2)	106 (88.3)	0.944
Would not object	4 (3.3)	5 (4.2)	
Not interested	9 (7.5)	9 (7.5)	
Do you agree with the following statements regarding getting your physician's email address? (scale of 1–5)			
It could improve the relationship between us:			
Mean ± SD	4.6 ± 0.69	4.58 ± 0.71	0.412
Range	2–5	2–5	
It could improve my sense of security even if I do not use it:			
Mean ± SD	4.59 ± 0.69	4.58 ± 0.71	0.456
Range	2–5	2–5	
Email is an effective means of communication that could solve my problems:			
Mean ± SD	4.33 ± 0.73	4.34 ± 0.72	0.457
Range	2–5	2–5	
Email can cut down on the number of clinic visits:			
Mean ± SD	4.38 ± 0.71	4.34 ± 0.72	0.667
Range	2–5	2–5	
Email can reduce the number of emergency room visits:			
Mean ± SD	4.38 ± 0.71	4.32 ± 0.75	0.500
Range	2–5	2–5	
At what times would you email the physician? [*N* (%)]			
I do not intend to call	9 (8)	9 (8)	0.994
Only at appointed hours	8 (7)	7 (6)	
Only during daytime hours (except Saturdays and holidays)	50 (42)	50 (42)	
At all hours including nights, Saturdays, and holidays	53 (44)	54 (45)	
Under which circumstance would you email your physician? [*N* (%)]			
I do not intend to contact by email	9 (8)	9 (8)	0.706
Only in unusual circumstances	44 (37)	38 (32)	
For any question	67 (56)	73 (61)	
The physician should not be sent an email because it could interfere with their privacy when they are not working (scale of 1 to 5):			
Mean ± SD	3.34 ± 1.42	3.41 ± 1.36	0.697
Range	1–5	1–5	
The physician should not be sent an email because medical errors can occur if a physical examination is not performed (scale of 1 to 5):			
Mean ± SD	3.88 ± 0.93	3.81 ± 0.96	0.566
Range	2–5	2–5	
The physician should not be sent an email because there is a risk of miscommunication (scale of 1 to 5):			
Mean ± SD	3.08 ± 0.85	4.03 ± 0.87	<0.0001
Range	2–5	1–5	
The physician should not be sent an email because it can interfere with their clinic work (scale of 1 to 5):			
Mean ± SD	3.20 ± 1.46	3.20 ± 1.47	1.000
Range	1–5	1–5	
I see no reason why I should not get the physician's personal email address (scale of 1–5):			
Mean ± SD	4.16 ± 0.92	4.39 ± 0.93	0.055
Range	1–5	1–5	
Have you asked for your physician's email address in the past? [*N* (%)]			
Yes	27 (23)	20 (17)	0.255
Do you have your physician's email address? [*N* (%)]			
Yes	73 (61)	46 (38)	0.0005
Have you contacted your physician by email since you became pregnant? [*N* (%)]			
Yes	41 (34)	23 (19)	0.009

**Table 4:** A comparison of attitudes towards receiving the family physician's cell phone number or email address.

Variable	Cell phone number	Email address	*P*
How do you feel about getting your physician's cell phone number or email address? [*N* (%)]			
Very interested	114 (95.0)	106 (88.3)	0.035
Would not object	5 (4.2)	5 (4.2)	
Not interested	1 (0.8)	9 (7.5)	
Do you agree with the following statements regarding getting your physician's cell phone number or email address? (scale of 1 to 5)			
It could improve the relationship between us:			
Mean ± SD	4.57 ± 0.71	4.58 ± 0.71	0.913
Range	2–5	2–5	
It could improve my sense of security even if I do not use it:			
Mean ± SD	4.58 ± 0.71	4.58 ± 0.71	0.913
Range	2–5	2–5	
Calls and email are effective means of communication that could solve my problems:			
Agree	4.37 ± 0.71	4.34 ± 0.72	0.745
Do not agree	2–5	2–5	
Calls and email can cut down on the number of clinic visits:			
Mean ± SD	4.33 ± 0.73	4.34 ± 0.72	0.915
Range	2–5	2–5	
Calls and email can reduce the number of emergency room visits:			
Mean ± SD	4.33 ± 0.74	4.32 ± 0.75	0.917
Range	2–5	2–5	
At what times would you call or email the physician? [*N* (%)]			
I do not intend to call or send an email	2 (2)	9 (8)	<0.0001
Only at appointed hours	49 (41)	7 (6)	
Only during daytime hours (except Saturdays and holidays)	54 (45)	50 (42)	
At all hours including nights, Saturdays, and holidays	15 (13)	54 (45)	
Under which circumstance would you call or email your physician? [*N* (%)]			
I do not intend to call or contact by email	1 (1)	9 (8)	<0.0001
Only in unusual circumstances	73 (61)	38 (32)	
For any question	46 (38)	73 (61)	
The physician should not be called or sent an email because it could interfere with their privacy when they are not working (scale of 1 to 5):			
Mean ± SD	4.03 ± 0.98	3.41 ± 1.36	0.0001
Range	1–5	1–5	
The physician should not be called or sent an email because medical errors can occur if a physical examination is not performed (scale of 1 to 5):			
Mean ± SD	3.73 ± 1.03	3.81 ± 0.96	0.876
Range	1–5	2–5	
The physician should not be called or sent an email because there is a risk of miscommunication (scale of to 5).			
Mean ± SD	3.98 ± 0.97	3.20 ± 1.47	<0.0001
Range	1–5	1–5	
The physician should not be called or sent an email because it can interfere with their clinic work (scale of 1 to 5):			
Mean ± SD	3.20 ± 1.46	3.20 ± 1.47	1.000
Range	1–5	1–5	
The family physician cannot help because I am pregnant: (scale of 1 to 5)			
Mean ± SD	1.98 ± 0.68	2.06 ± 0.74	0.384
Range	1–4	1–5	
I see no reason why I should not get the physician's personal cell phone number or email address (scale of 1 to 5):			
Mean ± SD	3.73 ± 1.05	4.39 ± 0.93	<0.0001
Range	1–5	1–5	
Have you asked for your physician's cell phone number or email address in the past? [*N* (%)]			
Yes	29 (24)	20 (17)	0.149
Do you have your physician's cell phone number or email address? [*N* (%)]			
Yes	50 (42)	46 (38)	0.598
Have you contacted your physician by cell phone or email since you became pregnant? [*N* (%)]			
Yes	26 (22)	23 (19)	0.631

**Table 5:** A comparison of attitudes towards receiving the gynecologist's cell phone number or email address.

Variable	Cell phone number	Email address	*P*
How do you feel about getting your physician's cell phone number or email address? [*N* (%)]			
Very interested	111 (92.5)	107 (89.2)	0.02
Would not object	8 (6.7)	4 (3.3)	
Not interested	1 (0.8)	9 (7.5)	
Do you agree with the following statements regarding getting your physician's cell phone number or email address? (scale of 1 to 5)			
It could improve the relationship between us:			
Mean ± SD	4.53 ± 0.78	4.60 ± 0.69	0.462
Range	2–5	2–5	
It could improve my sense of security even if I don't use it:			
Mean ± SD	4.53 ± 0.78	4.59 ± 0.69	0.528
Range	2–5	2–5	
Cell phone calls and email are effective means of communication that could solve my problems:			
Agree	4.28 ± 0.74	4.33 ± 0.73	0.598
Do not agree	2–5	2–5	
Cell phone calls and email can cut down on the number of clinic visits:			
Mean ± SD	4.28 ± 0.74	4.38 ± 0.71	0.286
Range	2–5	2–5	
Cell phone calls and email can reduce the number of emergency room visits:			
Mean ± SD	4.33 ± 0.74	4.38 ± 0.71	0.286
Range	2–5	2–5	
At what times would you call or email the physician? [*N* (%)]			
I do not intend to call	2 (2)	9 (8)	<0.0001
Only at appointed hours	47 (39)	8 (7)	
Only during daytime hours (except Saturdays and holidays)	56 (47)	50 (42)	
At all hours including nights, Saturdays, and holidays	15 (13)	53 (45)	
Under which circumstance would you call or email your physician? [*N* (%)]			
I do not intend to contact by email	1 (1)	9 (8)	<0.0001
Only in unusual circumstances	75 (63)	44 (37)	
For any question	44 (37)	67 (56)	
The physician should not be called or sent an email because it could interfere with their privacy when they are not working: (scale of 1 to 5)			
Mean ± SD	3.94 ± 0.86	3.41 ± 1.36	0.0001
Range	2–5	1–5	
The physician should not be called or sent an email because medical errors can occur if a physical examination is not performed: (scale of 1 to 5)			
Mean ± SD	3.67 ± 0.99	3.88 ± 0.93	0.091
Range	1–5	2–5	
The physician should not be called or sent an email because there is a risk of miscommunication: (scale of 1 to 5)			
Mean ± SD	3.92 ± 0.94	3.08 ± 0.85	<0.0001
Range	1–5	2–5	
The physician should not be called or sent an email because it can interfere with his clinic work: (scale of 1 to 5)			
Mean ± SD	4.00 ± 0.90	3.20 ± 1.47	<0.0001
Range	1–5	1–5	
I see no reason why I should not get the physician's personal cell phone number or email address: (scale of 1 to 5)			
Mean ± SD	3.62 ± 1.05	4.16 ± 0.92	<0.0001
Range	1–5	1–5	
Have you asked for your physician's cell phone number or email address in the past? [*N* (%)]			
Yes	33 (28)	27 (23)	0.371
Do you have your physician's cell phone number or email address? [*N* (%)]			
Yes	72 (60)	73 (61)	0.895
Have you contacted your physician by cell phone or email since you became pregnant? [*N* (%)]			
Yes	46 (38)	41 (34)	0.502

**Table 6:** Characteristics related to conduct Internet searches on pregnancy.

Variable	Often	Sometimes	Never	*P*
Age in years				
Mean ± SD	27.99 ± 3.91	27.58 ± 4.38	21.13 ± 2.59	<0.001
Range	19–35	19–37	18–24	
Family status [*N* (%)]				
Single	4 (6)	1 (2.2)	0	0.517
Married	63 (94)	44 (97.8)	8 (100)	
Place of residence [*N* (%)]				
Beer-Sheva	50 (74.6)	21 (46.7)	0	<0.001
Nearby city	9 (13.4)	9 (20.0)	2 (25.0)	
Agricultural settlement	6 (9.0)	5 (11.1)	0	
Bedouin sector	2 (3.0)	10 (22.2)	6 (75.0)	
Country of birth [*N* (%)]				
Israel	52 (77.6)	38 (84.4)	8 (100)	0.251
Other	15 (22.4)	7 (15.6)	0	
Years of education				
Mean ± SD	12.8 ± 0.55	11.76 ± 0.609	11.13 ± 1.126	0.001
Range	9–14	10–12	9–12	
Present work status [*N* (%)]				
Employed	54 (80.6)	28 (62.2)	0	<0.001
Student	9 (13.4)	5 (11.1)	2 (25.0)	
Unemployed	4 (6.0)	2 (25.0)	6 (75.0)	
Income				
Low	25 (37.3)	30 (66.67)	8 (100)	0.002
Average	31 (46.3)	12 (26.7)	0	
High	11 (16.4)	3 (6.7)	0	
How would you rate your health condition? [*N* (%)]				
Excellent	47 (70.1)	27 (60.0)	7 (87.5)	0.778
Very good	13 (19.4)	11 (24.4)	1 (12.5)	
Good	1 (1.5)	3 (6.7)	0	
Reasonable	5 (7.5)	3 (6.7)	0	
Poor	1 (1.5)	1 (2.2)	0	
Do you suffer from a chronic disease? [*N* (%)]				
Yes	7 (10.4)	5 (11.1)	0	0.617
No	60 (89.6)	40 (88.9)	8 (100)	
Population sector? [*N* (%)]				
Jewish	64 (95.5)	29 (64.4)	0	<0.001
Bedouin	3 (4.5)	16 (35.6)	8 (100)	
Are you religious? [*N* (%)]				
Yes	17 (25.4)	22 (48.9)	8 (100)	<0.001
No	50 (74.6)	23 (51.1)	0	
Number of children				
Mean ± SD	0.81 ± 0.925	1.53 ± 1.673	0.88 ± 1.126	0.013
Range	0–4	0–7	0–3	
Week of pregnancy				
Mean ± SD	20.55 ± 7.83	21.89 ± 8.359	17.75 ± 8.013	0.366
Range	7–37	7–36	11–35	
